# PROTOCOL: The promotion of well‐being among children exposed to intimate partner violence: a systematic review of interventions

**DOI:** 10.1002/CL2.216

**Published:** 2018-01-31

**Authors:** Natasha E. Latzman, Cecilia Casanueva, Julia Brinton, Valerie L. Forman‐Hoffman

## Background

### The problem, condition or issue

Children and adolescent's^1^ exposure to intimate partner violence (IPV), or domestic violence, is a pervasive public health problem. An estimated 8 to 15 million children in the United States (Hamby, Finkelhor, Turner, & Ormrod, 2011; McDonald, Jouriles, Ramisetty‐Mikler, Caetano, & Green, 2006) and 275 million children worldwide are exposed to IPV each year (Pinheiro, 2006). The consequences of exposure can be severe and long‐lasting. Research has linked IPV exposure in childhood to impaired neurological, physiological, and psychosocial functioning that contribute to a wide‐range of health consequences. Indeed, IPV exposure has been associated with reduced cognitive ability and educational achievement (Kitzmann et al., 2003), under‐immunization (Bair‐Merritt, Blackstone, & Feudtner, 2008), and both psychological (e.g., posttraumatic stress, depression, aggression; Davies, Evans, & DiLillo, 2008) and physical health problems (e.g., ischemic heart disease, obesity; Felitti et al., 1998). These negative developmental sequalae appear to be evident across nations and cultures; for example, the link between IPV exposure and future physical and/or sexual victimization has been found in studies conducted in the United States, China, South Africa, Colombia, India, Egypt, the Philippines, and Mexico; see Runyan, Wattam, Ikeda, Hassan, & Ramiro, 2002).

Documentation of the immense magnitude and burden of children's exposure to IPV has been met with an increased interest in the development of intervention strategies to protect this vulnerable population and promote well‐being. Interventions for children exposed to IPV were initially developed in the late 1980s and 1990s and predominately focused on provision of general support; they were available only in battered women's shelters or from agencies providing services to victimized women (see Graham‐Bermann & Hughes, 2003 for a review of early programming). More recently, theory‐driven psychosocial programs serving children exposed to violence have been developed and established in other venues (e.g., school‐based mental health clinics, outpatient psychotherapy settings). A recent scan identified 23 unique programs designed to improve outcomes for children exposed to IPV currently being implemented across the United States, with at least 8 of these programs having been subject to one or more rigorous evaluations, including randomized controlled trials (Chamberlain, 2014). To date, however, no systematic review has been conducted to synthesize the state of this burgeoning literature and provide recommendations for research and practice.

### The intervention

Our review is focused on psychosocial interventions where the primary or secondary aim is the promotion of child well‐being following exposure to IPV. Psychosocial interventions are defined broadly to include a wide variety of services that emphasize psychological and/or social factors rather than biological factors. Interventions may be psychological in nature, such as psychotherapies of various orientations (e.g., cognitive‐behavioural or interpersonal therapy) *and/or* social in nature (e.g., peer support services) (England et al., 2015). Importantly, interventions must involve provision of psychosocial services to the exposed child, the exposed child plus a caregiver(s), or only a caregiver(s). Studies must include outcome data on at least one child outcome to be included. Studies that report only caregiver outcomes will be excluded.

Interventions can occur in any setting, provided they include a psychological and/or social component; including but not limited to domestic violence shelters or service organizations, schools, outpatient clinics, criminal justice settings, or hospitals. The treatment modalities vary, including individual, family, or group‐based treatment. Below we provide example interventions in each of these categories.

*Individual intervention*. One‐on‐one treatment permits attention to individualized traumatic cues, distorted thoughts, and behavioral interactions. For example, Trauma‐Focused Cognitive Behavioral Therapy (TF‐CBT; Cohen, Mannarino, & Deblinger, 2006) is a clinic‐based intervention – rooted in learning and cognitive theories—addresses distorted beliefs and attributions related to the traumatic experiences and provides a supportive environment in which the child can talk about the traumatic experience or abuse. The treatment focuses on individual therapy sessions with children ages 3 to 18 years with parallel parent sessions that focus on the same elements as their child. Often, joint parent‐child sessions are also conducted. TF‐CBT is typically 12 to 16 sessions in length, although it has been modified into a shorter version for delivery in domestic violence shelters (Cohen, Mannarino, & Iyengar, 2011).

*Family‐based intervention*. Family‐based interventions, such as Child‐Parent Psychotherapy (CPP; Lieberman, 2004) involve sessions with a caregiver and a child age 0 to 5 years – with the dyad as the unit of treatment. CPP focuses on the focusing on the child–mother relationship as the therapeutic mechanism of change and usually delivered by therapists in 12 to 40 hour‐long sessions. Whereas CPP was designed to address exposure to trauma broadly defined, Project Support (Jouriles, McDonald, Rosenfield, Stephens, Corbitt‐Shindler, & Miller, 2009) was developed specifically for mothers and their children, age 4 to 9 years, with a history of IPV exposure. This family‐based intervention is delivered by a therapist in the mother's home and focuses on increasing the mother's problem solving and behaviour management skills. Project Support is typically involves 20 home visits over a six month period.

*Group intervention*. Group interventions, which typically are administered in schools, community settings, and domestic violence shelters, target general beliefs and attitudes about violence, reactions to violence, and social problem solving skills. For example, Kids' Club and Mom's Empowerment Program (MEP) (Graham‐Berman, 2000; Graham‐Bermann, Lynch, Banyard, DeVoe, & Halabu, 2007) are two group‐based psychosocial programs delivered in a broad range of settings (e.g., community‐based agency, outpatient mental health clinic). Kids' Club is designed for children age 6 to 12 years and creates a safe space for children to identify and express emotions and build social, emotional and coping skills. Moms Empowerment is a 10‐session parenting group that provides support to mothers of children age 6 to 12 years by empowering them to discuss the impact of the violence on their child's development and to build parenting competence.

### How the intervention might work

The potential pathway of effect between intervention and well‐being outcomes vary depending on the intervention – and specifically, the intervention's theoretical orientation.

For example, MEP has an interpersonal relationship orientation. Based in part on Sullivan's (1953) interpersonal theory, the MEP emphasizes the whole person and explores strengths and abilities that can be used to compensate for relative weaknesses or psychosocial dysfunction. The groups were designed to provide a venue for exploring relationships, and specifically, by telling their stories and histories of IPV victimization, connecting events to emotional reactions, and enhancing self‐esteem, it is theorized that levels of traumatic stress will decrease, with subsequent improvements in parenting and child well‐being (Graham‐Berman et al., 2007).

Interventions taking a cognitive‐behavioural orientation share the basic premise that psychological distress is maintained by maladaptive cognitions, or general beliefs about oneself and the world, contributing to specific and automatic thoughts in particular situations. The basic models holds that strategies to change these maladaptive cognitions leads to decreases in emotional distress and problematic behaviours and increases in well‐being. For example, TF‐CBT includes components teaching cognitive coping skills and cognitive restructuring (Cohen et al., 2006). Because at this point the full range of orientations is not known (we will search the full range of possible psychosocial interventions), we will outline theories of change for orientations and approaches that are included in the final review.

### Why it is important to do the review

Our **primary goal** is to systematically examine the available evidence for the effectiveness of psychosocial interventions for promoting well‐being following children's exposure to IPV. Here we use a broad definition of psychosocial interventions, as used by the Institute of Medicine of the National Academies, to include a wide variety of services (e.g., assessment, psychological counselling, group interventions, and education and support services that include a psychological and/or social component) that emphasize psychological and/or social factors rather than biological factors (England, Butler, Gonzales, 2015). Our **secondary goal** is to examine whether interventions with particular characteristics (modality, theoretical orientation, and setting) are more effective than others in promoting well‐being. Through this process, we aim to identify gaps in the current scientific literature and highlight important areas for future research to build the evidence base.

To date, no previous studies have synthesized the results of empirical evaluations of interventions designed to promote well‐being following exposure to IPV in childhood. Several literature scans, critical reviews and systematic reviews have been conducted in related areas, however. For example, Futures without Violence (Chamberlain, 2014) recently released a scan of interventions designed for children exposed to IPV. The scan was focused on programs based in the U.S., and the authors did not systematically review and synthesize the results of empirical evaluations. Wethington et al. (2008) reviewed a range of interventions intended to reduce psychological harm from traumatic events—broadly defined—among children, adolescents, and young adults. This review did not separate out children's exposure to IPV from other types of trauma exposures (e.g., car accidents). Further, in 2013, two systematic reviews were commissioned by the Agency for Healthcare Research and Quality (AHRQ) Effective Healthcare Program (both of which were co‐authored by researchers on the current proposal). In the first, Goldman and colleagues (2013) conducted a comparative effectiveness review of interventions addressing child maltreatment, and in the second, Forman‐Hoffman and colleagues (2013) conducted a comparative effectiveness review of interventions for children exposed to nonrelational traumatic events (e.g., accidents, natural disasters). Both of these reviews excluded evaluations of programs designed for children exposed to IPV, given the different nature of the trauma, resulting system responses, and intervention settings. For example, children exposed to IPV may not have access to resources traditionally offered to children engaged with the child protection system. Further, due to their own victimization, mothers experiencing IPV may be less able to provide support and stability to their own children (Lieberman & Van Horn, 2008); in fact, research indicates that mothers return to IPV perpetrators a mean of five times before permanently ending the abusive relationships (Sullivan & Bybee, 2011).

Results of this systematic review have practice and policy implications. Given the immense magnitude and burden of children's exposure to IPV, there is a need for intervention strategies to protect this vulnerable population and promote well‐being. It is critical that practitioners and policymakers are provided with evidence‐based recommendations on the most efficacious interventions. Researchers may also use this work to help shape future studies, including, for example, selection of programs to subject to rigorous outcome evaluations, or to extend to a new population.

## Objectives

Our review seeks to answer three main questions:

(1) Are psychosocial interventions targeting children who have been exposed to intimate partner violence (IPV) effective at promoting well‐being? Specific domains of well‐being include:
  a. Mental and behavioral health (e.g., posttraumatic stress symptoms, depressive symptoms; anxiety symptoms, adjustment problems, disruptive, aggressive, delinquent behavior, future victimization)
  b. Other development and school‐based functioning (e.g., cognitive development, academic achievement, social skills, executive functioning)
(2) Are interventions with particular characteristics more effective than others in promoting well‐being among children exposed to IPV? Specific intervention characteristics include:
  a. Modality (e.g., individual, family‐based)
  b. Theoretical orientation/approach (e.g., cognitive‐behavioral, interpersonal)
  c. Type of setting (e.g., domestic violence shelter, outpatient clinic).

## Methodology

### Criteria for including and excluding studies

#### Types of study designs

We plan to identify and assess experimental and quasi‐experimental designs where the primary or secondary aim is the promotion of well‐being following child exposure to IPV. Only studies that have a well‐defined control group will be included (e.g., wait‐list control, treatment‐as‐usual – such as a mother receiving supportive services at a shelter or a referral sheet). Designs may include:

‐ Randomized controlled trials in which individuals or parent‐child pairs are randomly assigned to intervention and comparison conditions;
‐ Quasi‐randomized controlled trials in which assignment to conditions is quasi‐random, such as by birth date, day of the week, or another alternation method;
‐ Controlled quasi‐experimental designs in which participants are not assigned to conditions randomly or quasi‐randomly, but in which participants self‐select into groups. Given the potential selection biases with controlled quasi‐experimental designs, we will only include the following types of quasi‐experimental designs: a) regression discontinuity designs; b) studies that use propensity score matching or other matching procedures to create intervention and comparison groups; or c) studies in which participants in the intervention and control groups are not matched, enough statistical information must be reported that will allow estimation of pre‐intervention effect sizes on at least one outcome.

Pre‐post designs without any comparison group and studies presenting only qualitative data will be excluded. Risk of bias will be assessed using the Cochrane Risk of Bias tool for each study (Higgins et al., 2008); the strength of evidence will be graded per criteria defined by Owens and colleagues (2010), which incorporates risk of bias, consistency, directness, and precision of the evidence for each outcome.

#### Types of participants

The specific population of interest is children and adolescents aged 0 to 17 years who have been exposed to IPV. Studies will be included if >75% of the study population is in the age range of 0 to 17 years.

Of note, we use a broad definition of exposure to IPV, guided by Holden's (2003) taxonomy of exposure and the Centers for Disease Control and Prevention's (CDC) definition of IPV (see, also, Latzman, Vivolo‐Kantor, Clinton‐Sherrod, Casanueva, & Carr, 2017):

- Exposure: Holden's (2003) comprehensive taxonomy of children's exposure to IPV outlines specific types of exposure, each falling into one of the four broad dimensions of prenatal exposure (e.g., mothers' perception that the prenatal IPV had effects on their fetus), direct involvement (e.g., child intervenes during the incident), direct eyewitness (e.g., child sees the incident) and indirect exposure (e.g., child is exposed to the aftermath such as helping a parent with injuries). Here we focus on the latter three types of exposure (and not prenatal exposure) due to our focus on IPV exposure experienced between the ages of 0 and 17.
- IPV: The CDC outlines four subtypes of IPV that can be perpetrated by a current or former intimate partner: physical violence (e.g., slapping, use of a weapon), sexual violence (contact and noncontact behaviours such as rape, sexual harassment), stalking, and psychological aggression (expressive aggression and coercive control) (Breiding, Basile, Smith, Black, & Mahendra, 2015).

We will exclude studies with children broadly identified as “at risk” for exposure (e.g., have experienced other types of victimization other than IPV exposure). Our review will be international in scope and we will not apply any restrictions related to nationality, language or cultural background, although we will code and consider study country/population's Human Development Index (HDI).

#### Types of interventions

We plan to include any type of psychosocial intervention where the primary or secondary aim is the promotion of child well‐being following exposure to IPV. Included interventions must involve provision of psychosocial services to the exposed child; psychosocial interventions are defined broadly to include a wide variety of services (e.g., assessment, psychological counselling, group interventions, and education and support services) that emphasize psychological and/or social factors rather than biological factors. Interventions may be psychological in nature, such as psychotherapies of various orientations (e.g., cognitive‐behavioural or interpersonal therapy) *and/or* social in nature (e.g., peer support services) (England et al., 2015). Interventions that focus services solely on the child's caregiver (i.e., services provided to only women at domestic violence shelters) will be excluded. However, we will include interventions that are multicomponent in nature and target both the child and risk factors related to the child's parent(s) or family environment. Interventions can occur in any setting, provided they include a psychological and/or social component; including but not limited to domestic violence shelters or service organizations, schools, outpatient clinics, or hospitals. For example, Kid's Club and Mom's Empowerment (Graham‐Berman, 2000) is a psychosocial program delivered in a broad range of settings (e.g., community‐based agency, outpatient mental health clinic) both within the U.S. and abroad. It includes both parenting programs and groups for children exposed to IPV that focus on building social, emotional and coping skills.

There are a number of interventions which are designed for children exposed to trauma, broadly defined (e.g., child maltreatment, witnessed community violence, car accidents). In these interventions, children may or may not have been exposed to IPV. For example, Cognitive‐Behavioral Intervention for Trauma in Schools (CBITS), is a school‐based psychosocial program delivered to children exposed to violence, *including but not limited to* intimate partner violence (Kataoka et al., 2013). If results are not reported for children who have been exposed to IPV (at least, but not limited to) then we will not include the study in our review.

#### Types of outcome measures

To prevent bias, outcomes measures will not be used as a criteria for inclusion in the review (Petticrew & Roberts, 2008). At the synthesis stage, only outcome measures addressing child well‐being will be analysed. Any program implemented with IPV‐exposed children aged 0 to 17 that intended to address these well‐being outcomes (whether as a primary or secondary outcome) will included in the present review.

- Mental and behavioral health (including but not limited to posttraumatic stress symptoms, depressive symptoms, anxiety symptoms, disruptive, aggressive, delinquent behavior, future victimization)
- Other development and school‐based functioning (including but not limited to cognitive functioning, academic achievement, and executive functioning)

#### Duration of follow‐up

All durations of follow‐up will be included.

#### Types of settings

All types of settings will be included.

### Search strategy

The search strategy will be developed in three stages. First, keywords derived from the study's inclusion criteria will be combined using Boolean operators and used to identify potentially relevant studies using the PsycINFO database (see Appendix I for this list). Citation chaining will also be used to identify additional relevant studies as well as those citing studies comprising the initial subset. Second, indexing of the identified citations will be examined for subject headings and relevant keywords capable of being added to terms of the search. Finally, the revised search strategy will be executed.

The assistance of a librarian with experience supporting systematic reviews will be sought in searching published, peer‐reviewed literature using validated search strategies for the following electronic databases: PubMed (Medline), PsycINFO, PsycARTICLES, Psychology and Behavioral Sciences Collection, Cumulative Index to Nursing and Allied Health Literature (CINAHL), Education Resource Information Center (ERIC), SocIndex with Full Text, Academic Search Premier, and ProQuest Dissertations and Theses. In addition, Google and Google Scholar will be used to identify literature not formally published in sources such as books or journal articles (i.e., grey literature) using a combination of keywords and outcomes of interest. Pubmed will also be searched for publications ahead of print. Databases will be searched from inception to the present date. Search results will be merged using EndNote and duplicate records of the same publication/report will be removed. We will contact investigators to retrieve papers for which only abstracts are readily available and will also contact experts within the field with queries regarding other potentially relevant studies of which they may be aware. Finally, the search employed in the review will be updated prior to submission for publication to ensure that the most recent data are captured.

Two independent reviewers will first independently screen studies' titles and abstracts. Potentially eligible studies – those marked as potentially include by either reviewer ‐ will then be retrieved in full text and these full texts will be reviewed for eligibility, again using two independent reviewers. Disagreements between reviewers will again be resolved via discussion and consensus, and if needed, consultation of a third study team member. If we cannot determine eligibility due to missing information, we will contact study authors for this information. The completed review will include a table of studies excluded at the full text level of screening and provide rationale for each exclusion decision. We will also publish a PRISMA flow chart to report the screening process and results (Moher et al., 2015).

Only studies that have a well‐defined control group will be included (e.g., wait‐list control, treatment‐as‐usual). Based upon Chamberlain's (2014) scan, we expect at least 8 randomized controlled trials with other studies employing quasi‐experimental designs.

### Criteria for determination of independent findings

In the case of multiple outcomes within a study, we will report the outcomes separately when possible (e.g., reporting depression and academic grades separately). When a single study reports results on multiple measures of the same outcome (e.g., two different measures of depression), we will select a single outcome based on a “pre‐defined hierarchy of outcomes” to reduce selection bias as suggested in the MEC2IR (2014) Standards. The elements of the hierarchy are that (a) we will choose parent (or other‐reporter, such as a teacher) for externalizing symptomatology (e.g., disruptive behaviours) and (b) youth‐report for internalizing symptomatology (e.g., depression). If element (a) is insufficient to make a decision about which of multiple measures of an outcome to include, (b) we will then choose the measure with the greatest reliability and validity coefficients. If element (b) is insufficient (e.g., there is a tie or no reliability or validity information is available) we will then choose the measure randomly. If there are multiple groups, only those findings from the control groups and intervention groups which meet the eligibility criteria will be included. However, the addition of another intervention group will be reported in the table presenting study characteristics.

### Details of study coding categories

For studies that meet our inclusion criteria, we will abstract important information into evidence tables. We will design data abstraction forms to gather pertinent information from each article, including characteristics of study populations, settings, interventions, comparators, study designs, methods, and results. Trained reviewers will extract the relevant data from each included article into the evidence tables. A second member of the team will review all data abstractions for completeness and accuracy. Coding disagreements will be resolved via discussion and consensus, and if needed, consultation of a third team member. If data needed to calculate an effect size are missing from a report, we will contact the primary study authors for this information.

The preliminary primary categories for coding are as follows:

- Publication characteristics
  ○ Publication type – article, book, dissertation, unpublished report
  ○ Year
- Participant demographics by group
  ○ Gender
  ○ Age/grade‐level
  ○ Race/ethnicity
  ○ Socioeconomic status
- Intervention characteristics by group
  ○ Geographic location
  ○ Setting (e.g., domestic violence shelter, school)
  ○ Orientation (e.g., cognitive‐behavioural)
- Risk of bias/Research Design
  ○ Random assignment
  ○ Type of design
  ○ Equivalence established by matching
  ○ Equivalence established by statistical controls (e.g., demographic characteristics, other child maltreatment experiences)
  ○ Equivalence established by Group sample sizes
  ○ Attrition bias
- Outcome construct
  ○ Type of construct
    ▪ Mental and behavioral health (e.g., posttraumatic stress symptoms, depressive symptoms; anxiety symptoms, adjustment problems, disruptive, aggressive, and delinquent behavior, victimization)
    ▪ Other development and school‐based functioning (e.g., cognitive development, academic achievement, social skills, executive functioning)
  ○ Measure used for each construct
    ▪ Reliability of measure
- Analyses
  ○ Information to compute effect sizes for each outcome
    ▪ For continuous outcomes: Mean, standard deviation, and *n* size of control group and treatment group
    ▪ For dichotomous outcomes: Number of events and number of possible events for control group and treatment group
    ▪ Other effect size information
  ○ Effect sizes based on raw or statistically adjusted data
  ○ Significance level for effect size
  ○ Direction of finding favors intervention group
  ○ Amount of missing data in percent
  ○ Dosage – number of sessions
  ○ Duration of follow‐up

The risk of bias in included studies will be assessed using predefined, design‐specific criteria based on guidance from the Agency for Healthcare Research and Quality (AHRQ)'s Methods Guide for Comparative Effectiveness Reviews. Two trained members of the study team will independently rate the risk of bias for each study; conflicts will be resolved by consensus or by consulting a third member of the team. In general terms, a study with no identifiable flaws will be rated as having low risk of bias. A study rated as having with medium risk of bias will be one deemed susceptible to some bias but probably not sufficient to invalidate its results. A study rated as having high risk of bias will have significant methodological flaws (stemming from, for example, serious errors in design or conduct) that may invalidate its results. We plan to consider the risk of bias for each relevant outcome of a study. When studies do not report sufficient detail to assess the validity of the design or study conduct, we will judge the risk of bias to be unclear.

### Statistical procedures and conventions

If we find two or more similar studies for a comparison of interest, we will conduct a meta‐analysis of the data from those studies. We will use random‐effects models to estimate pooled or comparative effects. In order to determine whether quantitative analyses are appropriate, we will assess the clinical and methodological heterogeneity of the studies under consideration following established guidance. We will do this by qualitatively assessing the different types of populations, interventions, comparators, and outcomes of each of the included studies, looking for similarities and differences. If we are able to conduct quantitative syntheses, we will assess statistical heterogeneity in effects between studies by calculating the chi‐squared statistic and the I^2^ statistic (the proportion of variation in study estimates due to heterogeneity). The importance of the observed value of I^2^ depends on the magnitude and direction of effects and on the strength of evidence for heterogeneity (e.g., p‐value from the chi‐squared test, or a confidence interval for I^2^). We will also examine potential sources of heterogeneity using sensitivity analysis or analysis of subgroups. We plan to stratify analyses and/or perform subgroup analyses when possible and appropriate to examine clinical heterogeneity. Planned stratifications or categories for subgroup analyses include the subgroups listed in the analytic framework and geographic location of studies. When quantitative analyses are not appropriate (e.g., due to heterogeneity, insufficient numbers of similar studies, or insufficiency or variation in outcome reporting), we will describe each study and its findings, separately.

Often times, studies that have multiple components involved in the intervention cannot be combined with other studies because of their complexity. In these instances, it is difficult to decipher which component or set of components in the intervention are responsible for the outcomes. If we decide that we want to investigate the efficacy of individual components of an intervention, we will use traditional variable‐oriented methods such as meta‐regression to deconstruct the unit of analysis into its component variables and then assess statistical correlations among one or more variables.

### Treatment of qualitative research

We do not plan to include qualitative research.

### Publication bias

We will assess publication bias by constructing a funnel plot to display the precision versus effect sizes of each included study.

## Review authors

**Lead review author:** The lead author is the person who develops and co‐ordinates the review team, discusses and assigns roles for individual members of the review team, liaises with the editorial base and takes responsibility for the on‐going updates of the review.

**Table:** 

Name: Natasha E. Latzman, PhD	
Title: Research Psychologist	
Affiliation: RTI International	
Address: 3040 Cornwallis Rd.	
City, State, Province or County: Research Triangle Park, NC	
Postal Code: 27709	
Country: USA	
Phone: 919‐541‐5884	
Email: nlatzman@rti.org	
**Co‐author(s):**	
Name: Cecilia Casanueva, PhD	** **
Title: Health Research Analyst	
Affiliation: RTI International	
Address: 3040 Cornwallis Rd.	
City, State, Province or County: Research Triangle Park, NC	
Postal Code: 27709	
Country: USA	
Phone: 919‐485‐2772	
Email: ccasanueva@rti.org	
**Co‐author(s):**	
Name: Julia Brinton, MA	** **
Title: Public Health Analyst	
Affiliation: RTI International	
Address: 3040 Cornwallis Rd.	
City, State, Province or County: Research Triangle Park, NC	
Postal Code: 27709	
Country: USA	
Phone: 919‐485‐5613	
Email: jbrinton@rti.org	
**Co‐author(s):**	
Name: Valerie L. Forman‐Hoffman, PhD, MPH	** **
Title: Senior Research Epidemiologist	
Affiliation: RTI International	
Address: 3040 Cornwallis Rd.	
City, State, Province or County: Research Triangle Park, NC	
Postal Code: 27709	
Country: USA	
Phone: 919‐485‐5625	
Email: vhoffman@rti.org	

## Roles and responsibilities

- Content: Natasha Latzman, Cecilia Casanueva
- Systematic Review Methods: Natasha Latzman, Valerie Forman‐Hoffman
- Statistical Analysis: Natasha Latzman, Valerie Forman‐Hoffman
- Information Retrieval: Natasha Latzman, Julia Brinton, Valerie Forman‐Hoffman

The team will be led by Principal Investigator *Natasha Latzman*, who has substantive expertise in violence and victimization, child and adolescent mental health, and evaluation research. She has training in systematic review methodology and will lead the project, including ensuring all milestones are met on time, developing the protocol, overseeing the literature retrieval, participating in analysis, and leading the development of the final paper. She will be supported by *Cecilia Casanueva*, an expert in IPV and child maltreatment, who will contribute to coding, synthesis and report writing; *Julia Brinton*, an experienced social science analyst, who will contribute information retrieval, database management, citation screening and coding; and *Valerie Forman‐Hoffman*, an expert in review methodology and statistical analysis, who serve as senior advisor, providing input on all stages of the review process, including data extraction, assessing bias and quality, and conducting analyses.

## Sources of support

We received funding under the Jacobs Foundation (in partnership with the Campbell Collaboration) call for proposals, *Better Evidence for Children and Youth*.

## Declarations of interest

None of the researchers involved in the team present conflicts of interest to note.

## Preliminary timeframe

Approximate date for submission of the systematic review: 20 October 2017

## Plans for updating the review

The lead reviewer anticipates updating the review on a five‐year cycle, pending continued research on the topic.

## AUTHOR DECLARATION

### Authors' responsibilities

By completing this form, you accept responsibility for preparing, maintaining and updating the review in accordance with Campbell Collaboration policy. The Campbell Collaboration will provide as much support as possible to assist with the preparation of the review.

A draft review must be submitted to the relevant Coordinating Group within two years of protocol publication. If drafts are not submitted before the agreed deadlines, or if we are unable to contact you for an extended period, the relevant Coordinating Group has the right to de‐register the title or transfer the title to alternative authors. The Coordinating Group also has the right to de‐register or transfer the title if it does not meet the standards of the Coordinating Group and/or the Campbell Collaboration.

You accept responsibility for maintaining the review in light of new evidence, comments and criticisms, and other developments, and updating the review at least once every five years, or, if requested, transferring responsibility for maintaining the review to others as agreed with the Coordinating Group.

### Publication in the Campbell Library

The support of the Coordinating Group in preparing your review is conditional upon your agreement to publish the protocol, finished review, and subsequent updates in the Campbell Library. The Campbell Collaboration places no restrictions on publication of the findings of a Campbell systematic review in a more abbreviated form as a journal article either before or after the publication of the monograph version in Campbell Systematic Reviews. Some journals, however, have restrictions that preclude publication of findings that have been, or will be, reported elsewhere and authors considering publication in such a journal should be aware of possible conflict with publication of the monograph version in Campbell Systematic Reviews. Publication in a journal after publication or in press status in Campbell Systematic Reviews should acknowledge the Campbell version and include a citation to it. Note that systematic reviews published in Campbell Systematic Reviews and co‐registered with the Cochrane Collaboration may have additional requirements or restrictions for co‐publication. Review authors accept responsibility for meeting any co‐publication requirements.

**I understand the commitment required to undertake a Campbell review, and agree to publish in the Campbell Library. Signed on behalf of the authors**:

**Table:** 

**Form completed by: Natasha E. Latzman**	**Date: April 7, 2017**

## Footnotes

1 Hereafter “children” is used to refer to children and adolescents aged 0 to 17 years.

## Appendix

Three categories of keywords will be used for the search. The first category lists key terms related to the population of interest: youth. The second category addresses exposure to IPV. The third category aims to identify “evidence focused” papers. In case the search capacity is limited only the term ‘evaluation’ will be used as it is the most sensitive. Some databases allow choosing ‘RCT’ as publication type, in which case the search will be run twice with this option included. The intention of separating the terms in this manner is to include all the potentially relevant results, while simultaneously excluding the large bodies of literature on the impact of adult and youth exposure to IPV.

Our review will cover a broad‐range of outcomes related to well‐being, spanning mental and behavioral health to cognitive and school‐based functioning. Therefore, specifying key words related to well‐being will likely be too limiting and exclude studies that omit well‐being information from the title and abstract. Instead, specific outcomes will be retrieved and coded during full‐text review.

These three sets of keywords will be combined with a Boolean AND.

**Table:**

**Age Group:**	Child* OR Youth OR Teen* OR Minor OR Kid OR Juvenile OR Adoles*
	OR Toddler OR Baby
**Exposure:**	(Expos* OR Witness* OR Observe OR hear* OR see*) AND (Fight* OR Viol* OR Aggress*
	OR assault) AND (Partner OR Domestic OR Parent* OR Family OR
	Mother OR Boyfriend OR Girlfriend)
**Outcome:**	Evaluation OR Empirical OR Experiment* OR Trial OR Effective* OR Efficacy

The final search terms will be described in detail in the full review so that it can be fully and precisely replicated.
